# Decision frameworks for restoration & adaptation investment–Applying lessons from asset-intensive industries to the Great Barrier Reef

**DOI:** 10.1371/journal.pone.0240460

**Published:** 2020-11-03

**Authors:** Mayuran Sivapalan, Jerome Bowen

**Affiliations:** Decision and Risk Advisory, Aurecon, Melbourne, Australia; University of Defence, SERBIA

## Abstract

Asset-intensive industries (including water and power utilities, mineral resources and energy) are those which require significant levels of capital investment in their assets in order to operate. These industries face challenges from uncertainty in resource availability and demand for end products, the intricate and complicated nature of their assets, and the complexity of the economic, ecological and social settings in which they operate. In these industries, the application of decision frameworks that account for this uncertainty and complexity in guiding asset investment and development is standard practice. Lessons from asset-intensive industries were applied during the concept feasibility phase of the Reef Restoration and Adaptation Program (RRAP) to establish the investment case for research and development into interventions to help the Great Barrier Reef (GBR) resist, adapt to, and recover from the impacts of climate change. The authors worked with RRAP partners to define a decision framework that included structured decision-making processes (SDM), a cost-benefit analysis (CBA), and a value of information (VoI) analysis, to establish the investment case for intervening on the GBR which led to success in securing Australian Government commitment for the next phase of the Program. With climate change expected to drive increased demand for significant levels of restoration and adaptation investment in large integrated social, ecological and economic assets (such as the GBR), the lessons from RRAP offer insights for the application of decision frameworks to inform public and private investment priorities.

## Introduction and background

### The challenge facing the Great Barrier Reef

The Great Barrier Reef (GBR) is the world’s largest living structure, an ecosystem home to a wealth of marine biodiversity unmatched anywhere in the world, a global icon so exceptional that it has been inscribed on the United Nations (UN) World Heritage List since 1981 in recognition of its Outstanding Universal Value [1].

In 2014, the Great Barrier Reef Marine Park Authority (GBRMPA) identified deterioration in key habitats, species and ecosystem processes from the cumulative effects of declining water quality from land-based runoff, marine pests and cyclones [2]. More recently, GBRMPA identified the risk posed by climate change, specifically increases in sea temperatures leading to coral bleaching, ocean acidification and increasingly frequent and severe weather events, as the most significant threat to the GBR’s long term health [3,4]. There has been very little rigorous scientific investigation of the prospects for at-scale reef restoration and adaptation. The methods that have been used at small scales are not suitable for larger-scale efforts and the scale of the scientific endeavour has been inconsistent with the magnitude and complexity of the challenge [5]. For coral reefs to survive and prosper in a likely warmer future, investment in new interventions to support reef restoration and adaptation are urgently needed [5].

### The Reef Restoration and Adaptation Program

The Reef Restoration and Adaptation Program (RRAP) brings together world-leading experts to help the GBR resist, adapt to, and recover from the impacts of climate change. It aims to provide reef managers with a toolkit of innovative interventions to be implemented at scale, through comprehensive research and development (R&D). An initial 18-month, AUD 6 million concept feasibility phase of RRAP was tasked with examining the overall feasibility of undertaking restoration and adaptation interventions to protect key ecological functions and economic and social values of the GBR.

### Establishing the investment case

Over the first 12 months of this concept feasibility phase, work progressed to mature understanding of the relevant issues and uncertainties in the assessment of the feasibility, costs, risks and benefits of the numerous solutions and techniques proposed. With 6 months of the concept feasibility phase remaining and a need to begin developing the investment case, the following had been established: 1) the extent of uncertainty in the resilience and response of ecological processes to climate change scenarios, across spatial and temporal scales, 2) confirmation of the myriad uncertainties that impacted intervention development, assessment and selection, including and not limited to intervention costs, maturity, efficacy and deployment scale, 3) the extent of uncertainty in understanding of values across ecological, economic and social dimensions, and 4) challenges associated with a deep and heterogeneous knowledge base across stakeholder, regulatory, engineering, ecological, social and economic dimensions. These issues and uncertainties posed a challenge to the Program in being able to establish defensible costs and benefits associated with interventions and thus successfully establish an investment case for the Program.

### Decision frameworks in asset-intensive industries

Given these challenges, the potential applicability of decision-making approaches used successfully in complex asset-investment decision contexts, to focus the activities and outputs of the concept feasibility phase towards the investment case, was established.

Asset-intensive industries (for example, water and power utilities, mineral resources and energy) are those which require significant levels of capital investment in their physical assets in order to operate, usually preceded by a high-cost research and development stage. These industries face challenges from 1) uncertainty in resource availability and market demand for end products, 2) the intricate and complicated nature of their assets, and 3) the complexity of the economic, ecological and social settings in which they typically operate. In these industries, it is standard practice to apply frameworks drawn from decision science that account for this uncertainty and complexity in guiding asset investment and development. Examples of the application of decision frameworks and structured decision-making processes can be found in the energy sector [6–9], the minerals sector [10] and water and power utilities [11–13], as well as in other large industries where the theory and practice is progressed (the defense sector [14–16] and the pharmaceutical industry [17,18]).

The concept of decision frameworks emanates from operations research and decision science, which comprise a suite of processes and tools used to inform managerial decision-making. By focusing on decisions as the fulcrum between the potential solutions to problems and the action taken to solve them, operations research and decision science provide approaches for characterising complex problems, and for identifying, assessing and committing to solutions to those problems. For this reason, analytical processes and tools from operations research and decision science are used extensively in complex asset-intensive industries to inform decision-making. The science underpinning operations research and decision analysis has been summarised in *Decision Analysis*: *An Overview* [19] from the detail provided in multiple publications [20–29].

The common challenges that impact the establishment of investment cases in assets, in both asset-intensive industries and for large integrated social, ecological and economic assets such as the GBR include 1) complexity of the asset, 2) disparate and heterogenous information streams, 3) need for multi-functional / multi-disciplinary stakeholder input and engagement, 4) uncertainty in asset performance in the face of external pressures and threats and 5) preponderance of potential solutions.

The application of approaches from operations research and decision science to large integrated social, ecological and economic assets such as the GBR have gained momentum in recent years. In the GBR specifically, they had previously been identified as having potential utility in helping support the decision-making needs of GBR managers [30,31], and has recently been applied directly on decisions for the GBR in helping establish and prioritise monitoring and reporting efforts [32]. There are also examples of application to environmental issues in small community contexts [33], and on a preliminary scale to coral reef ecosystems [34,35].

Compared to these earlier examples, the application of approaches from operations research and decision science described in this paper is novel as it 1) is focussed on a strategic decision (as opposed to tactical), 2) describes the application of multiple decision support methods and tools as part of an overall decision framework (as opposed to a single method) and 3) is a practical application (as opposed to a theoretical application) to establish an investment case that has since been funded.

The question being asked during the concept feasibility study was: *Are the approaches used to inform decision-making on complex problems in asset-intensive industries useful for informing decision-making on complex problems in large*, *integrated*, *social*, *ecological and economic assets*?

## Decision science processes and methods

**Fig 1:**
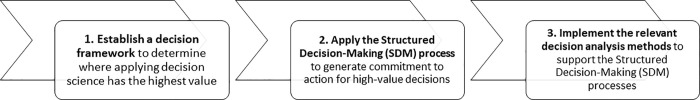
The three-stage approach taken on RRAP to apply lessons from asset-intensive industries to decision-making on the GBR.

The approaches were designed to test the application of decision frameworks from the asset-intensive industries to investment in restoration and adaptation on the Great Barrier Reef (GBR.) A three-stage approach was used to identify, develop and execute the activities required to inform the investment case for RRAP. This three-stage approach is summarised below in Fig 1. Each stage is dependent on a clear understanding of the *decision framework* for the program.

### Establish a decision framework

#### Decision framework

**Fig 2:**
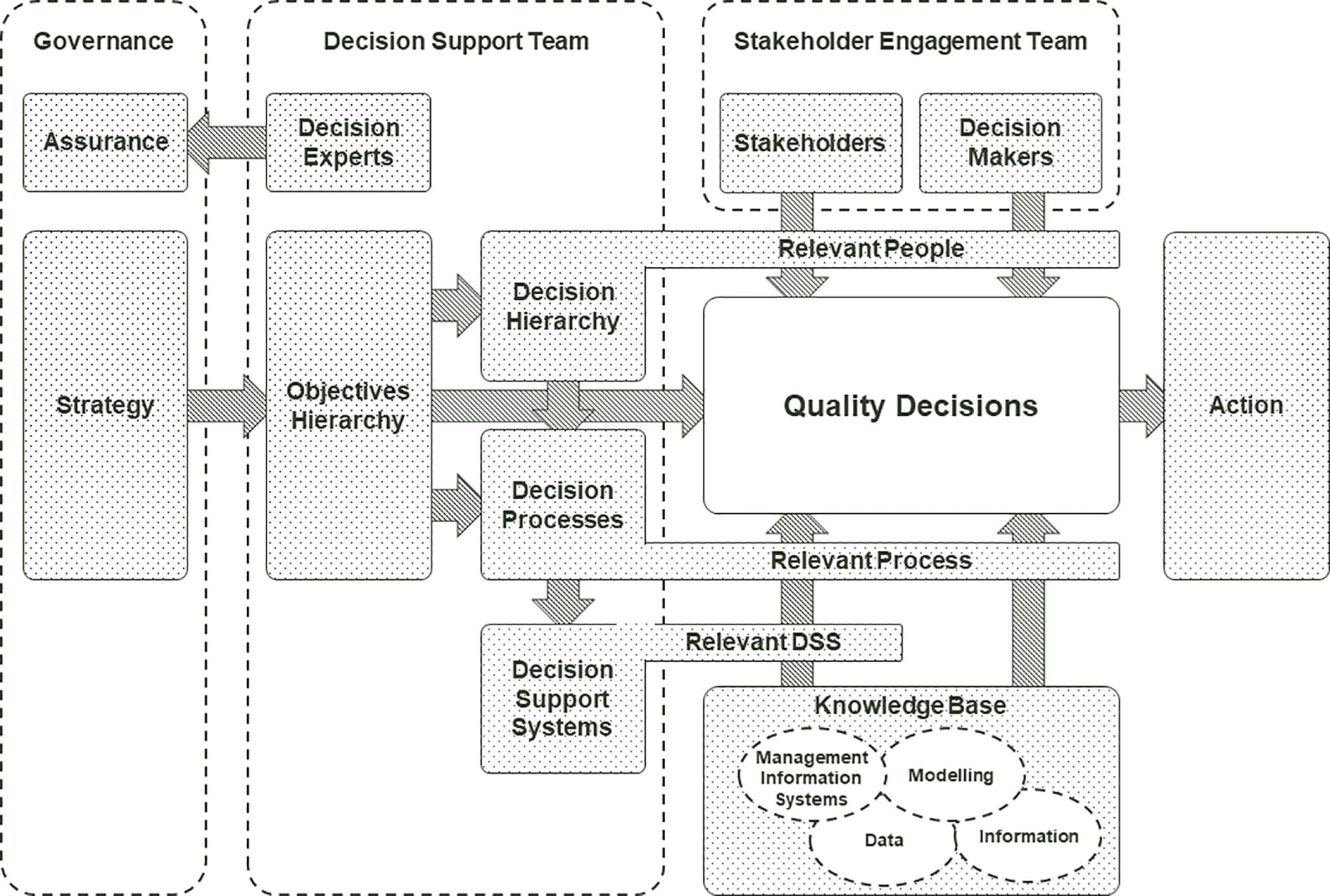
Visualisation of a comprehensive decision framework to be applied to the RRAP objectives and decision space.

Decision frameworks are required to characterise the interaction between the people, processes and systems for decision-making about a complex asset. Decision frameworks existing in multiple settings and can be described as the principles, processes, and practices to proceed from information and desires to choices that inform actions and outcomes [31]. The visualisation of a comprehensive decision framework in Fig 2, developed by the authors, draws on this literature, as well as our view of best-practice from our experience in asset-intensive industries. This is the theoretical framework through which the RRAP complications were proposed to be addressed. Each of the core elements of this decision framework are described in more detail below. The definition used in this scope for a decision framework is:

*“Decision Framework*: *The architecture of people*, *processes and systems used to make decisions*”.

#### Decision hierarchy

For RRAP we first established the **decision hierarchy** which is a key element of a coherent decision framework in large, complex assets. They are used in the early phase of large developments and in the management of operating large assets to characterise and organise problems and opportunities according to the potential value proposition of successful solutions and the complexity and uncertainty that impacts identification and selection of potential solutions. Decision hierarchies are used in asset management contexts [36], business case development [37], software development for decision-makers [38], and in theory of decision-making [39]. The definition of decision hierarchy used by the authors draws on this literature, as well as our view of best-practice definition from our experience in asset-intensive industries, and is defined as below:

*“Decision Hierarchy*: *The organisation of the decisions that need to be made*, *supported by who makes those decisions and how they relate to objectives*, *value drivers and boundaries across the decision landscape”*.

#### Objectives hierarchy

The setting of a coherent **objectives hierarchy** is critical to the success of any project or program that would benefit from implementing a decision framework. They are used in sustainability contexts [40], in contexts where asset developers are trying to balance financial and broader organisational aspirations [41], where public agencies are seeking to develop social infrastructure [42], and numerous applications are presented in the earliest decision science literature [25]. The definition of objectives hierarchy used by the authors draws on this literature, as well as our view of best-practice definition from our experience in asset-intensive industries, and is defined as below:

*“Objectives Hierarchy*: *The arrangement of organisational*, *program and / or project objectives into relevant hierarchical levels”*.

The objectives hierarchy should be derived directly from the **strategy** for the program as set by the appropriate governance body. The **decision support experts** serve the dual role of providing subject matter expertise to support relevant processes and activities that inform decision making, and, of providing **assurance** for the governance group ensuring there is an effective link between decision-making and strategy.

#### Decision processes

The **decision processes** used by the program are instrumental to successful alignment of stakeholders and thus to executing quality decisions. The importance of effective business processes is well understood in management circles [43] and in the decision sciences [19,28,44]. Clearly understanding and planning decision processes, as well as identifying the relevant processes for each decision in the decision hierarchy will maximise the chance that decision-making is smooth and aligned. The definition of decision processes used by the authors is as below:

*“Decision Processes*: *The processes by which decisions are framed*, *choices are identified*, *developed and logically analysed for their consequences and trade-offs*, *and commitments to action are made*, *across the decision landscape”*.

#### Stakeholder engagement

The importance of **stakeholder engagement** in a decision framework is paramount as alignment among the relevant people, using the right processes and tools, and the right knowledge, is necessary for quality decisions. A key part to this is ensuring the relevant stakeholders are prepared to undertake the discussions required to commit to action. There is strong evidence throughout decision science and management literature that stakeholder engagement and participation is key to decision-making success [45].

#### Knowledge base, management information systems and decision support systems

Decision processes require an appropriate **knowledge base** upon which to make the decisions. This includes accessing the right modelling, data and information, as well as the use of relevant **management information systems** and **decision support systems** where appropriate. Ensuring that the best possible knowledge is accessible to decision-makers is an important aspect in the development and management of large asset bases [12,13,46,47] and is described variously in dedicated journal publications (e.g., Decision Support Systems Journal). The availability of knowledge through management information systems and the utility of decision support systems are characterised widely in operations research, decision science and information management literature [48–52]. The definition of knowledge base, management information system and decision support system used by the authors are as below:

*“Knowledge Base*: *The data*, *modelling and information available pertaining to the asset*, *including understanding of provenance and uncertainty*, *used to inform decision-making*”.

*“Management Information System*: *The computerised system that gathers data from multiple sources and makes it available to users (including synthesis) to support quality decision-making*”.

*“Decision Support System*: *The computerised system that gathers data from identified sources*, *synthesises it*, *and makes it available to users in accordance with specified decision processes to support quality decision-making on specific semi-structured and unstructured decision problems*”.

#### Quality decisions

The comprehensive decision framework is brought together by the requirements for **quality decisions**, which include effectiveness, transparency, defensibility, consistency, efficiency and an understanding of certainty. Each of these requirements maps to a function within the decision framework for research or development programs as shown below in Table 1; for example, *effectiveness* maps to the *objectives hierarchy* through *outcomes*, and *consistency* maps to the *decision processes* through *process*. These requirements are described variously in theoretical texts for decision sciences [53,54].

**Table 1:** Requirements of a comprehensive decision framework for a research or development program, including how they map to key decision framework functions.

Requirement	Informs	Function
Effectiveness	Outcomes	Objectives Hierarchy
Transparency	Engagement	Stakeholder Management
Defensibility	Alignment	Decision Hierarchy
Consistency	Process	Decision Processes
Efficiency	Prioritisation	Value of information
Certainty	Effort	Work Breakdown

#### Value of information and work breakdown

There are two decision framework functions that serve as **key planning interfaces** with the wider research and development program in which it sits. The decision framework requirement for *efficiency* is made functional through **value of information (VoI)** method that will help with *prioritising* where effort could be spent in the rest of the program to decrease uncertainty in the knowledge base–this method is detailed later in this paper. The decision framework requirement for *certainty* is made functional through a **work breakdown** of the rest of the program that focuses *effort* on decreasing uncertainty in the knowledge base as prioritised by the decision framework. As such, a comprehensive decision framework should help the program managers to effectively *prioritise effort* to *efficiently increase certainty* to inform higher quality decisions.

### Applying the structured decision-making process

Structured decision-making (SDM) is a general term to describe approaches used to help individuals and groups navigate through tough multi-dimensional choices characterised by uncertain science, disparate information, diverse stakeholders and difficult trade-offs [44]. SDM is derived from the science of decision analysis, the foundations of which are summarised in *Decision Analysis*: *An Overview* [19]. The primary purpose of SDM is to aid and inform decision-makers, rather than to prescribe a preferred solution. It is based on the concept that quality decisions are those which are based on values (understanding what’s important) and consequences (understanding what’s likely to happen). It is aimed at providing consistency, transparency and defensibility to decisions.

SDM is particularly helpful for complex problems, like restoration and adaptation on the GBR, where, multiple disciplines (e.g., engineering, environmental science, social science and finance) need to work together to develop solutions that are rigorous, inclusive, defensible and transparent [44]. The factors that contribute to a decision problem being complex are presented in Table 2. Because of these factors, complex decisions can be characterised as being high stakes, complicated, having no single overarching expert and requiring justification. The decisions in RRAP meet each of these criteria.

**Table 2:** Characteristics of Complex Decision Problems compared to the Reef Restoration and Adaptation Program (RRAP).

Attribute	RRAP	Supporting Comment
The existence of multiple objectives and drivers of value	Yes	RRAP aspirational objective is “healthy reef, healthy people”
Good alternatives being difficult to immediately identify	Yes	Interventions considered in RRAP have never been done before at scale
Consequences extend over long time-horizons	Yes	Climate change impacts extend in perpetuity; timescales of coral recovery from bleaching and cyclones are decadal [5]
Consequences and outcomes subject to significant uncertainty	Yes	Novel interventions, uncertainty in biological processes and economic value
Multiple Impacted Groups	Yes	Complex socio-ecological space
Trade-offs between multiple value	Yes	Self-evident
No single decision-maker	Yes	Board and steering committee ultimate arbiter of decisions

**Fig 3:**
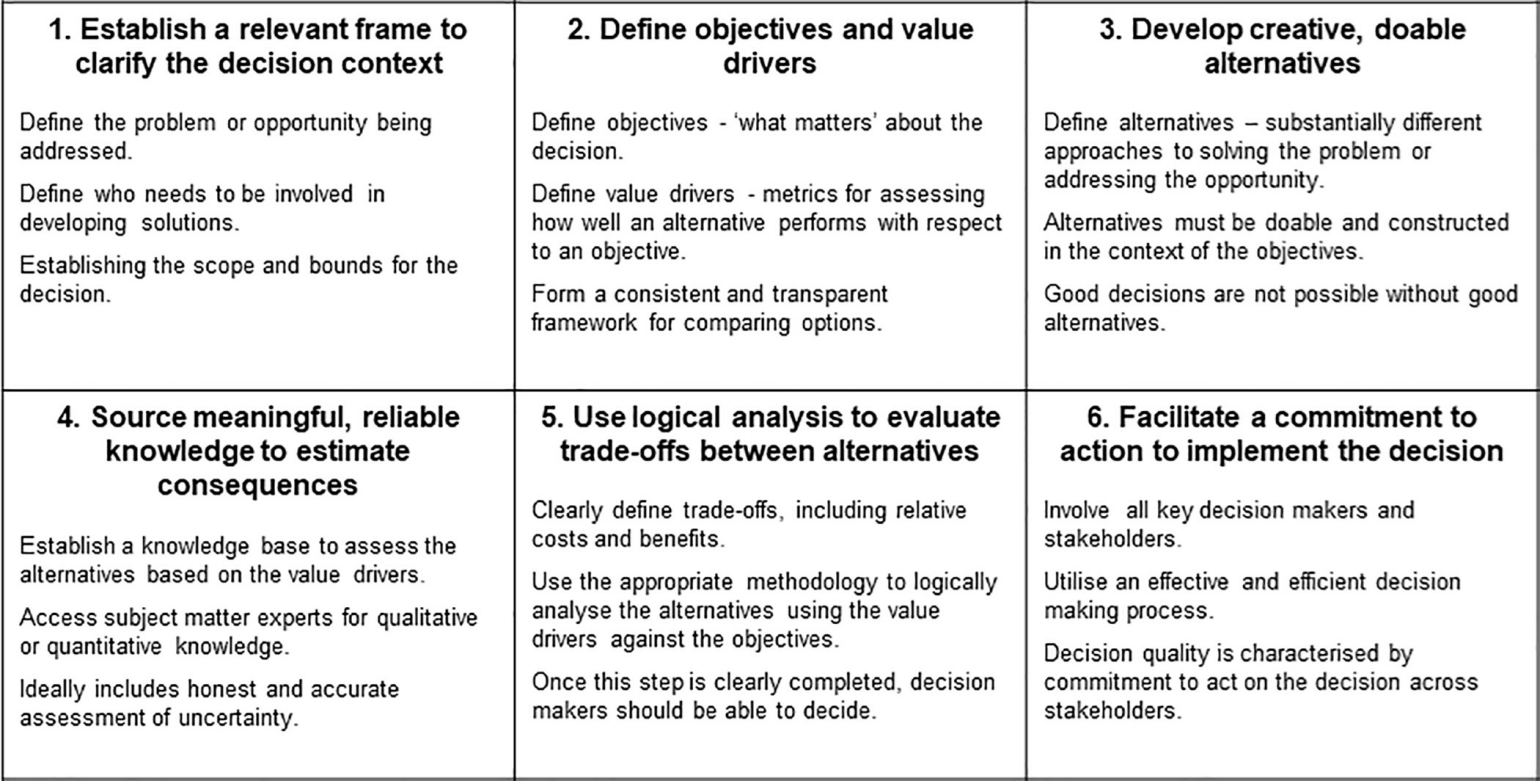
The six (6) core steps of structured decision-making applied during the Reef Restoration and Adaptation Project (RRAP). Adapted from: [44].

While there are numerous variations of SDM processes used to support complex decision-making [55], almost all share the six core steps that were used in this project as summarised below (Fig 3).

### Implementing the relevant decision analysis methods

#### The cost-benefit analysis method

This section summarises the cost-benefit analysis (CBA) method, with a more detailed method available in *Reef Restoration and Adaptation Program*: *Cost-Benefit Analysis* [56].

CBA was identified as a potentially relevant analysis method to establish and communicate the value of intervention. CBA attempts to systematically estimate and compare the total benefits and costs of a proposal by calculating the dollar value of the gains and losses for all people affected. CBA is typically used by governments (including the Australian Government who is the ultimate target funder of the RRAP) as (1) it provides decision makers with quantitative and qualitative information about the likely effects of a proposal, (2) encourages decision-makers to take account of all the positive and negative effects of a proposal, and discourages them from making decisions based only on the impacts on a single group, (3) is typically expressed in a single unit ($) which promotes comparability and assists in the assessment of relative priorities across proposals and (4) captures linkages between the proposal and other sectors of the economy, especially when proposals are large in scale such that wider economic effects are material.

The CBA method that was proposed for the scope of work involved producing an economic model for assessing the benefits of environmental and social protection against the costs of the restoration and adaptation required to do so. The method is based on those presented in *The Economics of Groundwater Remediation and Protection* [57], and *Environmental and Economic Sustainability* [47], and is aligned to standard cost-benefit analysis processes used by governments globally [58–60]. The approach describes and measures the case for investment in economic terms, by explicitly monetising costs and benefits to society to the extent possible and appropriate, and adding these to private costs and benefits of a proposed project or action.

In economics, the overall objective of any decision is assumed to be the maximisation of human welfare over time. To compare the different benefit and cost streams over time, the process of discounting is used and amounts over time are expressed as “present values”. Economic analysis recommends the decision with the maximum net present value (NPV present value of net benefits, or benefits minus costs, over time) or the highest benefit cost ratio (ratio of the present value of benefits to the present value of costs).

In practice, only some of the benefits identified can be readily quantified and monetised, but those considered in the cost-benefit analysis must be sufficient to determine the case for investment. Those monetised are likely be several of the key private benefits (such as benefits to industry, benefits to livelihoods). External benefits (such as benefits to communities, benefits to ecosystems) are less readily monetised as there is often no market data that could be directly used for their estimation.

#### The value of information method

This section summarises the value of information (VoI) method, with a more detailed method available in Appendix C of *Reef Restoration and Adaptation Program*: *Cost-Benefit Analysis* [56].

Value of information (VoI) is a field of decision analysis used to estimate the value of acquiring additional data to reduce decision uncertainty. The origins of VoI lie in the work carried out by Raiffa and Schlaifer on applied statistical decision theory at Harvard University in 1961 [61]. It has had many applications in different decision-making domains including, medical, economic, environmental, agricultural and ecological research [62–64]. Whereas basic decision analysis enables decision-makers to identify the best course of action when faced with a situation of uncertainty, VoI provides guidance on how decision-makers might invest in reducing that uncertainty before selecting a course of action [62].

The starting point is some objective function to be maximised, and a choice between courses of action leading to uncertain outcomes with respect to the objective function [63]. In the case of RRAP the objective function is the intervention strategy, the course of action is implementation with or without additional research into these interventions, and the outcome is the resulting cost and efficacy of the intervention.

VoI can be described as the increase in expected value (and certainty) in the outcome of a course of action with the benefit of additional information relating to this action, compared to a course of action without the benefit of the same information. Alternatively, positive VoI occurs when the value in the outcome of a decision benefiting from additional information, minus the cost of obtaining that information, exceeds the value in the outcome of the same decision without the information.

On this basis, VoI can be used as a quantitative method to estimate the return on investment of carrying out additional research into an objective function (intervention), through some form of data collection exercise, with a view on increasing expected value (and certainty) in the outcome of the course of action chosen (implementation). The value of effort to increase certainty in current knowledge (e.g., research, expert elicitation, technology trials, field surveys, etc) can be assessed through this methodology and is referred to as pre-posterior analysis [62,63]. A common way to reduce uncertainty is to gain new information, however, not all uncertainty is equally important to reduce. The most important uncertainties are those that, when reduced, will enable a more effective solution and potentially lower cost and higher benefit. New information is of little value if it does not change the efficacy or cost of the proposed action in the absence of said new information [64].

VOI was identified as a potentially relevant analysis method to establish and communicate the value of research and development given (1) level of uncertainty in RRAP, (2) availability of the proposed Program budget and potential Program benefits from the CBA to inform a VOI analysis and (3) stated Program desire to prioritise effort in reducing uncertainty in the following stage of RRAP.

## Findings

### Decision hierarchy elicitation enables identification of and alignment on evolving high-value decisions

Eliciting and agreeing a decision hierarchy with a broad range of Program participants and stakeholders was found to be useful for RRAP, because as the program evolved, the highest value decision on which to focus RRAP efforts evolved and could be transparently communicated and understood. The process also enabled delineation of the decisions that were within the boundaries of RRAP, on the boundaries of the Program and outside the remit of the Program.

Decision framework processes such as framing workshops proved valuable in establishing the decision hierarchy for RRAP. In multiple workshops with key RRAP members, statements of success for RRAP were determined, which enabled identification of the highest value decision in this phase of the Program which needed to be the subject of the investment case. These workshops elicited, reviewed and refined a set of nine statements completing the sentence “we know we will be successful if…” (Table 3). These statements provided a detailed understanding of the requirements that RRAP leadership have for effectively communicating and justifying their decision-making outcomes both within and outside RRAP. These statements of success continued to be relevant throughout successive framing sessions for other high-value decisions throughout the Program.

**Table 3:** Nine statements of success were identified to inform the decision-making hierarchy for RRAP, as defined at the commencement of the engagement.

Statements of success *“We know we will be successful if…”*	
A	Stakeholders are confident that all the relevant inputs are captured that allow us to **articulate the value of the interventions** in RRAP.
B	We can take the outputs from ecological models and **effectively convert them into expression of value**; with an acceptable degree of rigour; and that all assumptions are considered reasonable / well sourced.
C	Decision outcomes are **accepted as capturing the range of opinions** of stakeholders; and all stakeholders are accepting of the decision outcomes.
D	The decision **appropriately makes use of the data available** at the correct level (i.e., sufficient resolution data in order to make the decision.)
E	The **decision process is adaptable** to future changes to the option set, value functions and valuations and is able to be used at various points over the RRAP process (six years).
F	The decision can **be informed with imperfect data** (with disclaimers).
G	Non-specialists and **non-familiar people** with reef modelling **can understand** how the decision accounts for uncertainty.
H	We can demonstrate that, from a cost-benefit perspective, **active restoration and adaptation interventions are a valid new management strategy** for the Great Barrier Reef (GBR.)
I	We can **effectively decide the relative effort among the intervention strategies** to be developed / researched over the next five years.

The highest-value decision identified for the purpose of establishing the investment case was “**What are the optimum Reef restoration and adaptation intervention strategies to invest in and deploy**”. However, it soon became clear that the high degree of uncertainty within RRAP meant that it was premature to be able to effectively characterise and assess the choices for this decision within the timeframe of the concept feasibility phase. This was a pattern throughout the study–the highest value decision as basis for the investment case evolved a further 3 times as understanding of the uncertainty in the Program increased. The timeline is presented in Table 4. The first decision is, however, likely to be the primary decision for subsequent phases of the Program as research activities decrease uncertainty.

**Table 4:** Identification order of four high-value decisions in the RRAP concept feasibility phase, including their level in the objectives hierarchy, their decision-making process, method and outcome.

ID order	Objectives Hierarchy Level	Decision	Process, Method, Outcome
1^st^ identified	0	“**What are the optimum Reef restoration and adaptation intervention strategie**s to invest in and deploy, that represent the greatest value proposition [including Reef 2050 values], given the expected costs of R&D and deployment and the range of risks and uncertainties around intervention strategies and climate change scenarios”?	SDM, CBA, discontinued
3^rd^ identified	1	“**How to prioritise R&D activities** that have the highest likelihood of contributing to the RRAP Objectives, taking into account the ranges of risks and uncertainties?”	SDM, MODA, discontinued
2^nd^ identified	2	“**How investable is RRAP** Research & Development program, given the expected costs of deployment and the range of risks and uncertainties within intervention strategies and climate change scenarios?”	SDM, CBA, completed
4^th^ identified	2	“What is the **inherent value of the RRAP Research & Development phase**, where the total value of RRAP including implementation (research, development and implementation) is communicated?”	SDM, Value of information, completed

The establishment of a decision hierarchy for RRAP was found to be an effective process in focussing decision-science effort on the highest value decisions possible to be answered given the available information (and uncertainty in information) to inform the investment case. The nature of this concept feasibility phase included many completely new areas of enquiry, large amounts of uncertainty, complex interfaces between disciplines, and evolving decision-making needs. The focus on understanding decision hierarchy at all times allowed for several effective pivots of the elements within the decision framework to follow adjustments in the Program due to this complexity, stimulated by the increased breadth and maturity of knowledge emerging from the Program’s social-scientists, scientists, engineers and modellers.

### SDM was the relevant process for RRAPs highest value decisions

It was found that structured decision-making (SDM) is a relevant process to be applied to RRAP as the attributes of complex decision problems match the attributes of RRAP. The SDM process was applied to four high-value decisions within RRAP, with two of those instances delivering value to the program objectives for the concept feasibility phase. The other two applications of SDM were found not to be effective at the concept feasibility phase as there was insufficient certainty of knowledge to commit to action; the SDM process is, however, highly likely to be applicable to these problems in later phases of the Program with greater availability of knowledge.

Details of how the SDM process was applied to four high-value decisions within RRAP is summarised in Table 5.

**Table 5:** High-level detailing of the structured decision-making (SDM) processes carried out during the RRAP concept feasibility phase.

Structured Decision-Making Process Step	What are optimum Reef intervention strategies?	How to prioritise R&D activities?	How investable is RRAP?	What value of the RRAP R&D program?
Establish a relevant frame	Successful, later determined to be **too broad** given uncertainty in knowledge	Successful	Successful	Successful
Define objectives and value drivers	Successful, later determined to be **too broad** given uncertainty in knowledge	Successful	Successful	Successful
Develop creative, doable alternatives	Successful	Successful	Successful	Successful
Source meaningful, reliable knowledge	**Too uncertain** to provide decision-making clarity	**Too uncertain** to provide decision-making clarity	Successful	Successful
Use logical analysis to evaluate trade-offs	Cost-benefit analysis (CBA)	Multi-objective decision analysis (MODA)	Cost-benefit analysis (CBA)	Value of information (VoI)
Facilitate a commitment to action	None, **process discontinued**	None, **process discontinued**	Considered investable, **next phase funding approved**	Considered valuable, **next phase funding approved**

The SDM processes were effective in aligning stakeholders, decision-makers and information sources from within the complex team to establish the investment case for the program. Two of the decisions will be detailed in the next section:

- Cost-benefit analysis (CBA) method to establish and communicate the value of intervention
- Value of information (VoI) method to establish and communicate the value of research and development

### Cost-benefit analysis is an effective method to establish and communicate the value of intervention

It was found that cost-benefit analysis (CBA) is a relevant method to communicate the value of intervention on the GBR, and by extension whether it is worth investing in RRAP. This section summarises the application of CBA during the concept feasibility phase and the relevant findings on the applicability of CBA method to RRAP. Detailed accounts of the CBA methodology are available in *Reef Restoration and Adaptation Program*: *Cost-Benefit Analysis* [56] and *Reef Restoration and Adaptation Program: Intervention Analysis and Recommendations [65].*

**Fig 4:**
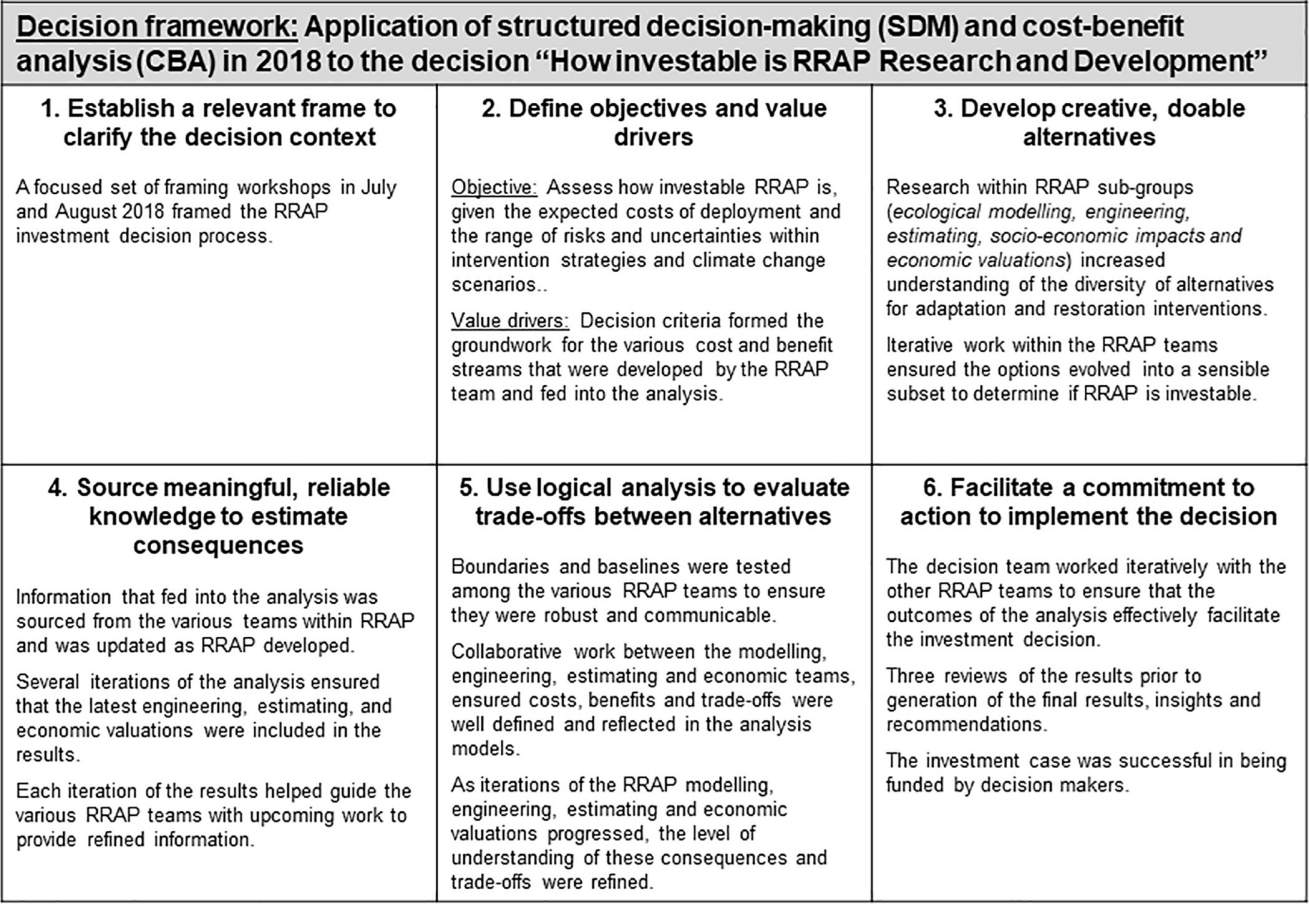
The six (6) core steps of structured decision-making applied to the decision “How investable is RRAP research and development”.

The intent of the CBA for RRAP was to demonstrate, within the high degree of uncertainty inherent in the program, whether there is a strong set of intervention options and conditions within which investment in RRAP is favourable, and thus enable decision-makers to determine whether the Program receives funding to progresses to the next stage. Simply, the analysis examines whether RRAP demonstrates enough potential net benefits to continue. The SDM process was used as the framework to guide the CBA, according to the stages summarised in Fig 4.

#### Key insights from the CBA

The CBA was effective in being able to establish that RRAP is an investable proposition. By taking into account the balance of costs and benefits, it was shown that there is significant potential economic upside from RRAP interventions of up to AUD 4.1B net present value (NPV) (2016 dollars, 3.5% discount rate) under base case assumptions, which is equivalent to AUD 28B undiscounted over 60 years. Taking a 10% exceedance probability for 1,000 iterations of sensitivity parameters, the potential upside net benefit from RRAP was shown to be up to AUD 14.5B NPV (2016, 3.5%). Thus, RRAP is likely an investable proposition for further R&D more often than not across a broad range of conditions and potential uncertainties. For more detailed presentation and discussion of these findings, please refer to the reports at the top of this section.

From a cost-benefit perspective, restoration and adaptation intervention is a valid new management strategy for the GBR and further R&D should be invested in. The CBA for RRAP showed that, within the high degree of uncertainty inherent in the Program, there are a strong set of conditions where active restoration and adaptation interventions are likely to deliver positive net benefits. As a result, it was recommended that the Program receive investment to progress to the next stage of R&D.

#### Key insights and recommendations for using CBA as a decision method for RRAP

The high degree of uncertainty within RRAP at many levels, at least one order of magnitude greater than what would typically be observed during a R&D program concept feasibility phase, means that this CBA should be considered an early attempt to examine the performance of potential program elements. That is, the assessment considered the high degrees of uncertainty within RRAP and examined, within this range, the potential futures for RRAP performance. It did not assume that engineering and scientific action will be taken that result in poor performance; it instead flags where further research will need to be undertaken to reduce uncertainty, better define potential performance, and thus ensure that only well-performing program elements are progressed.

The CBA process should be iterated as uncertainty decreases. It is one tool within RRAP that can be used to ensure only well performing program elements are advanced to the next stage. This informed high-level implications for the next stages of RRAP investment. However, it must be noted that the high degree of uncertainty within the source data means they are necessarily high-level. The SDM process that provided the framework for the CBA, however, is repeatable in subsequent RRAP stages to inform intervention selection and ensure optimised implementation of the Program.

### Value of information is an effective method to establish and communicate the value of research and development

Used in conjunction with the CBA, it was found that value of information (VoI) analysis is a relevant method to communicate the value of R&D into interventions on the GBR, and thus whether it is worth investing in the next R&D phase of RRAP. This section summarises the application of the VoI analysis during the concept feasibility phase and findings on the applicability of VoI methods to RRAP. Detailed accounts of the VoI methodology are available in *Reef Restoration and Adaptation Program: Cost-Benefit Analysis [56].*

**Fig 5:**
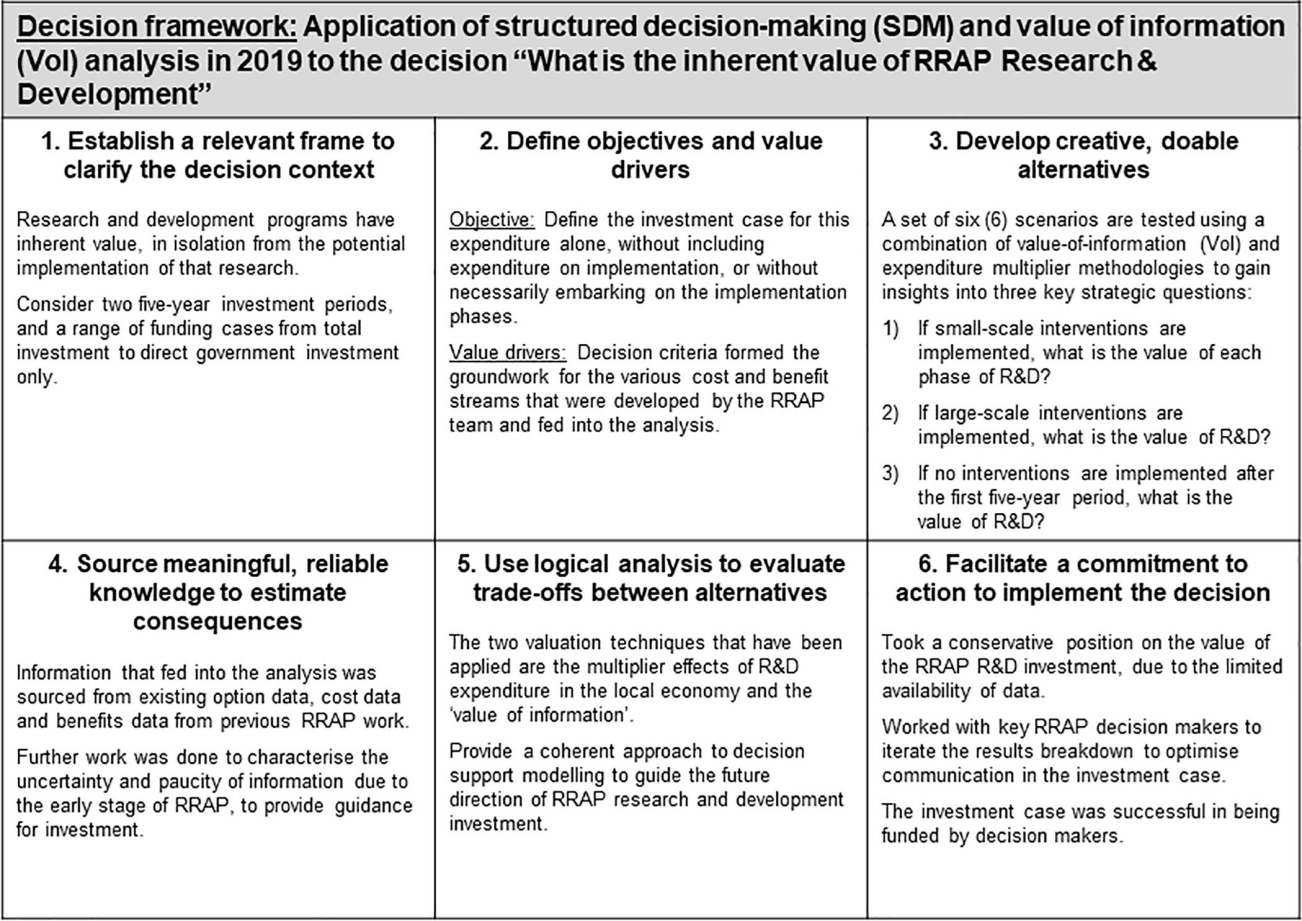
The six (6) core steps of structured decision-making applied to the decision “What is the inherent value of RRAP research & development?”.

R&D programs have inherent value, in isolation from the potential value from implementation of that R&D. To this end, we employed an SDM process to examine the inherent value of RRAP R&D. The stages are summarised in Fig 5.

#### Key insights from the value of information analysis

The VoI analysis was able to establish that there is a strong case for the investment in at least one phase of R&D, despite the conservative assumptions made in recognition of the significant uncertainty in the Program. The major insights that were established through the VoI analysis were:

- *Doing nothing (no R&D or implementation) is an option*, *but not one recommended by RRAP—*the CBA established that action on the GBR has significant potential upside of between AUD 4.1B and AUD 144B net present value (NPV) (2016, 3.5 percent discount rate), and conducting the R&D phase of the Program inherently retains this as an option value.
- *Attempting to deploy interventions without an R&D phase is not recommended—*the limited number of interventions that might be possible (i.e., technically feasible and able to gain regulatory approval without R&D) are small-scale, likely to be expensive per unit area, poorly guided, with high ensuing ecological risk and with no guarantee they would deliver net benefits, so R&D is essential to improve these factors.
- *Investing AUD 100m in an initial phase of R&D is highly likely to be worthwhile–*only a 10 percent improvement in the certainty of a small-scale intervention implementation being successful is required in order to justify the costs of the phase of R&D.
- *Securing an additional AUD 50m for an initial phase of R&D is highly likely to be worthwhile*–if success certainty increases by more than an additional 1.8 percent, the additional investment is worthwhile.
- *If implementation does not proceed after five years’ R&D*, *there remain positive benefits of the investment in R&D–*the benefits include expenditure multiplier, and research and knowledge benefits in the Australian economy. Quantification of the expenditure multiplier demonstrates that these benefits alone exceeds the cost of investment under reasonable assumptions.

#### Key insights and recommendations for using value of information as a decision method for RRAP

The successful application of VoI to establish and communicate the value proposition of investment in R&D provides a template for guiding both later phases of R&D investment, and, prioritising investment in program areas to resolve uncertainty during each phase of R&D. For the former, while a high likelihood of success of small-scale intervention after a second round of R&D is required to deem the second round R&D investment worthwhile, this decision point is four years away, allowing time for better data and information to generate more accurate insights prior to decision-making. For the latter, the ability of this approach to characterise the impact of uncertainty resolution on the ultimate net benefits of the program, enables prioritisation on uncertainty reducing activities in the program, and screening of those interventions where the cost of uncertainty reduction is incommensurate with the potential benefits.

## Implications and recommendations for the application of decision frameworks to establish the investment case for solutions to complex problems in large integrated social, ecological and economic assets

The findings from this application of decision frameworks commonly used in asset-intensive industries to establish the investment case for R&D in restoration and adaptation on the Great Barrier Reef (GBR) has implications for RRAP and for investments in large, integrated social, ecological and economic assets more broadly, especially in the context of climate change:

- Decision frameworks comprising processes and tools from decision science have high utility for generating commitment to action for large-scale restoration and adaptation research, development and intervention.
- Decision hierarchies, their determination and iteration, have high utility in R&D programs subject to high levels of uncertainty to enable rapid pivoting of the application of decision processes and tools to the highest value decisions.
- Structured decision-making (SDM) processes have high utility in R&D programs to generate quality decisions and commitment to action, especially in highly complex settings such as large social, ecological and economic assets with many stakeholders, requiring high levels of investment, and, with impacts of uncertain future scenarios such as climate change.
- The cost-benefit analysis (CBA) method has high utility in R&D programs to articulate the investment case of intervention to preserve large, integrated social, ecological and economic assets.
- The value of information (VoI) method has high utility in R&D programs to articulate the investment case for ongoing R&D into intervention options for large, integrated, social, ecological and economic assets.

Based on the outcomes of this application, and drawing upon the experience of the authors with similar applications in asset-intensive industries, the following recommendations are made for RRAP, and for other programs looking to establish the case for investment in restoration and / or adaptation R&D or intervention in similarly large, integrated, social, ecological and economic assets:

- Programs should consider implementing or upgrading to a comprehensive program-wide decision framework that enables integration of sub-program areas that feed knowledge and a characterisation of uncertainty into program planning and investment prioritisation processes.
- Programs should consider applying a coherent objective setting process, integrated with governance groups and strategic processes, that ensure alignment between program decision-making and desired strategic outcomes that can be transparently articulated through investment cases.
- Programs should consider establishing a decision hierarchy, ideally during conception, to map the decisions that need to be made, and by who, and how they relate to objectives, value drivers and boundaries across the program, to enable the highest value investment case to be made.
- Programs should ensure that asset knowledge (data, modelling and information) includes understanding of provenance and uncertainty, to enable investment cases to be framed according to the value proposition despite uncertainty, and, to present the investment case in terms of the value proposition of reducing uncertainty.

## Conclusions

This paper has demonstrated that the application of frameworks commonly used in asset-intensive industries, drawn from decision science, were critical in establishing the investment case for R&D in restoration and adaptation on the Great Barrier Reef (GBR) in the context of climate change. The resulting commitment from investors to fund the R&D phase of RRAP has resulted in the largest-ever coordinated action to assist a globally significant social, ecological and economic asset in its fight to survive climate change.

There is significant further work required in this area, below are some proposed areas of future research that could build on the findings in this paper:

- Extend the value of information (VoI) analytical approach, conducted at the program scale, to establish an iterative VoI process *within* RRAP to assist with prioritising investment in R&D.
- Extend the cost-benefit analysis (CBA) conducted at the program scale, to establish an iterative CBA process *within* RRAP to assist with prioritising investment in implementation of restoration and adaptation options.
- Characterise a decision framework for the entirety of RRAP, in order to streamline activities towards quality decision-making (that enables efficient, effective, transparent and defensible decisions.)
- Map knowledge and uncertainty within the decision framework for RRAP to enable quality decisions to be made about investing in reduction of uncertainty / improvement of knowledge.
- Investigate more widely the application of coherent decision frameworks than just the restoration and adaptation efforts on the GBR.
- Investigate more widely the application of coherent decision frameworks for informing decision-making on complex problems in large integrated social, ecological and economic assets with many stakeholders.

There are also many interfaces with other areas of research that afford ongoing debate, including:

- Is the monetization of benefits valid enough to justify investment cases for large scale adaptation and restoration, given the uncertainties of translating potential social and environmental values to monetary benefits?
- Do decision frameworks sufficiently characterise the social and political realities of stakeholder decision-making to have utility for investment decision-making?
- How are traditional owner perspectives and value systems appropriately represented through structured decision-making (SDM) processes?
- Are there thresholds whereby the extent of underlying uncertainty in knowledge areas significantly challenges the robustness of investment case recommendations?

## Limitations

There are several limitations in the application of decision processes and tools described in this paper. These should be considered when examining the results and implications presented.

- The high degree of uncertainty within RRAP at many levels of the program means that the application of the cost-benefit analysis (CBA) method should be considered an early attempt at examining the performance of potential program elements, and not as a demonstration of what will be implemented, nor a determinant of where implementation investments will ultimately be made. The analysis provides only high-level insights into which interventions could be invested in.
- The testing of sensitivity presented in this analysis is restricted by what was carried through in source information. This analysis has used source data from other RRAP teams that contains averaged or most likely assumptions or cases, meaning that sensitivity to those assumptions or cases is not demonstrable within these results.
- Economic valuation is restricted to defendable benefits, with likely total benefits being higher. The Program was unable to fill all gaps in understanding benefits at the concept feasibility phase. Similarly, only large-scale (Great Barrier Reef-wide and regional) costs and benefits have been assessed as the Program primarily examined large-scale interventions during this phase.
